# DMSH-Net: Depth-aware multi-scale hybrid vision network for image dehazing

**DOI:** 10.1371/journal.pone.0352586

**Published:** 2026-08-04

**Authors:** Chenping Zhao, Jun Li, Yingjun Wang, Zuhua Guo, Xiaoyue Li

**Affiliations:** 1 School of Computer Science and Technology, Henan Institute of Science and Technology, Xinxiang, China; 2 School of Computer Science and Technology, Henan Institute of Technology, Xinxiang, China; Shanghai Jiao Tong University, CHINA

## Abstract

Single-image dehazing remains a challenging low-level vision task because haze degradation is inherently depth-dependent and spatially non-uniform. To address this problem, we propose DMSH-Net, a Depth-Aware Multi-Scale Hybrid Vision Network specifically designed for robust single-image dehazing. DMSH-Net is designed to implicitly capture haze variations through hierarchical feature recalibration, nonlinear residual refinement, and multi-scale contextual aggregation. Specifically, we introduce a redesigned convolutional squeeze-and-excitation attention (CSEA) module, which replaces fully connected transformations with convolutional operations and global average pooling to jointly model channel dependencies and spatial context. Building on CSEA, a nonlinear CSEA-coupled residual block (NCCRB) is developed to enhance local feature representation and improve adaptability to haze with varying densities. Furthermore, a multi-scale dilated convolution bottleneck is incorporated to enlarge the receptive field and aggregate haze-aware contextual information across multiple spatial scales, thereby improving the restoration of regions with varying scene depths. Extensive experiments on standard benchmarks demonstrate that DMSH-Net consistently achieves superior quantitative performance across full-reference and no-reference evaluations, thereby validating its robustness in complex real-world dehazing scenarios.

## Introduction

As a fundamental problem in image restoration, single-image dehazing aims to recover clear scene information from fog-degraded observations, improving visual quality and realism [1]. With the increasing demand for all-weather vision in applications such as autonomous driving [2,3], drone inspection [4,5], smart agriculture [6,7], and remote sensing [8,9], robust dehazing has become particularly important. However, under hazy conditions, atmospheric scattering causes nonlinear attenuation of scene radiance, leading to low contrast, missing details, and colour distortion [10–12]. These degradations reduce the reliability of downstream tasks such as semantic segmentation and object detection [13–19]. Therefore, it is essential to remove haze while preserving textures, edges, and structural details.

Traditional dehazing methods mainly rely on the atmospheric scattering model [10,11,20,21], which is commonly written as:


$$\begin{array}{c} J(x)=I(x)\cdot t(x)+A\cdot (1-t(x)), \end{array}$$ (1)

where *J*(*x*) is the observed hazy image, *I*(*x*) is the haze-free image, $t(x)=e^{-\beta d(x)}$ is the transmission map, *d*(*x*) is the scene depth, and *A* denotes the global atmospheric light. The exponential form of *t*(*x*) reflects the strong nonlineari*t*y of haze, especially in regions with large depth or density variations. Based on the atmospheric scattering model in (1), the dark channel prior (DSP) method was proposed and developed [10,22,23], which estimated the transmission map *t*(*x*) based on the assumption tha*t* haze-free images contain low-intensity values in at least one color channel. These prior-based approaches have achieved relatively promising results to a certain extent. However, their reliance on hand-crafted assumptions often results in inaccurate transmission estimation, leading to inadequate performance under complex conditions.

With the rapid development of deep learning, convolutional neural networks (CNNs) have been widely applied to image dehazing [24–29]. End-to-end CNN frameworks [24,29,30] directly learn the mapping from hazy to clear images, alleviating the need for explicit estimation of transmission and atmospheric light. Multi-scale designs enhance contextual modeling and feature representation, while attention mechanisms [26,28,30,31] such as channel attention, spatial attention, and self-attention improve feature selection and global context modeling. These strategies have significantly advanced dehazing performance. Nevertheless, several challenges still remain. First, haze distribution often varies significantly with scene depth, and accurately modeling such spatial non-uniformity is still difficult [32]. Second, many channel attention modules that rely heavily on fully connected layers may ignore spatial context, weakening the preservation of fine textures. Last but not the least, conventional residual blocks lack sufficient flexibility to adapt to diverse haze patterns, particularly in complex or highly variable scenes.

In this work, we present DMSH-Net, a depth-aware multi-scale hybrid vision network for single-image dehazing, motivated by the depth-dependent and spatially non-uniform nature of haze degradation. In hazy scenes, regions at different depths typically suffer from varying degrees of contrast attenuation, texture degradation, and residual haze, making it essential to jointly consider local detail restoration and multi-scale contextual perception. Therefore, the proposed network leverages spatial context and multi-scale receptive fields to implicitly capture depth-related haze variations without introducing an additional depth estimation branch. To this end, DMSH-Net organizes feature recalibration, residual refinement, and multi-scale context aggregation into a unified framework. Specifically, we design a convolutional squeeze-and-excitation attention (CSEA) module that replaces fully connected layers with convolutional operations and global average pooling, thereby jointly capturing channel dependencies and spatial context to produce haze-sensitive feature recalibration. Building on this, a nonlinear CSEA-coupled residual block (NCCRB) integrates nonlinear activations with residual learning, enabling stable local feature refinement and improved texture preservation under varying haze densities. At the bottleneck, a multi-scale dilated convolution module further aggregates contextual information across multiple receptive fields, allowing the network to effectively perceive and handle haze variations from near to distant regions. Through this hierarchical and complementary design, DMSH-Net achieves robust quantitative performance, improved structural restoration, and strong generalization across synthetic and real-world dehazing benchmarks. The main contributions of this work are summarized as follows:

- We propose DMSH-Net, a depth-aware multi-scale hybrid network that models spatially non-uniform haze caused by scene depth variations, achieving robust dehazing while preserving edges and fine textures.
- We design an improved CSEA module and a nonlinear CSEA-coupled residual block (NCCRB) that jointly model channel dependencies, spatial context, and nonlinear local features, enhancing representation capacity and detail retention.
- We employ a multi-scale dilated convolution NCCRB bottleneck to capture depth-aware haze variations across multiple receptive fields, and demonstrate through extensive experiments and ablations that this design yields consistent gains on both synthetic and real-world benchmarks.

## Related work

### Model-based methods

Restore clear images by simulating the impact of haze on images. Although these methods provide some theoretical bases, their effectiveness is often limited by difficult-to-obtain parameters such as environmental illumination and viewing distance, and they perform poorly in complex scenarios. To overcome these problems, more studies have begun to attempt deep learning-based methods [33]. Early single-image dehazing research was mainly based on prior knowledge and used it to estimate the transmission map and atmospheric light value. He et al. [10] proposed a dark-channel prior. It assumes that most non-sky regions in haze-free images have some pixel values with low intensity in at least one channel. Zhu et al. [22] in 2015 proposed a color attenuation prior. They analyzed a large number of blurred images and observed a positive correlation between the blurring concentration and the depth of field. Berman et al. [20]proposed a non-local prior algorithm, which assumes that hundreds of colors can approximate the colors of haze-free images. However, in most previous studies, when the complexity of the actual environment does not meet these priors, the atmospheric light value and the transmission map are often inaccurately estimated. This can significantly affect the dehazing effect. Recent prior-based methods, such as IHDCP [34], further revisit haze-density correction for single-image dehazing.

### Deep learning-based methods

Automatically extract features from images through end-to-end learning, thereby avoiding the complex computational steps in physical models [10,33]. Li et al. [25] proposed an integrated network for end-to-end image dehazing by reformulating the atmospheric scattering model. Liu et al. [26] proposed a GridDehazeNet, which consists of preprocessing, a backbone, and postprocessing. In GridDehazeNet, a multi-scale estimation based on an attention-based grid network is used to achieve a robust dehazing effect. The reverse projection pyramid network proposed by Singh et al. [35] has also achieved remarkable results in the dehazing task. However, these CNN-based methods still have deficiencies in feature extraction and multi-scale feature fusion, especially in complex scenes where image details are prone to loss or excessive smoothing. Moreover, the training stability of deep networks is poor, which can easily lead to gradient vanishing or explosion, affecting the convergence and dehazing effect of the model. Recent physical-prior-guided transformer methods, such as SwinTD-Net [36], have also been introduced to enhance haze-aware representation and image restoration.

In addressing these issues, the introduction of attention mechanisms, residual convolutional networks, and multi-scale parallel dilated convolutional kernel modules provide new ideas [37]. Hu et al. [31] introduced global average pooling through a channel attention mechanism to generate channel weights, although there are problems of spatial information loss and parameter redundancy. Wang et al. [38] proposed using one-dimensional convolution instead of fully connected layers to reduce the number of parameters. Chen et al. [27] introduced a gating mechanism, which enhanced the feature extraction ability but increased training instability. In image restoration tasks, residual convolutional networks such as ResNet and RDN can alleviate the problem of gradient vanishing but still face issues of high-frequency detail smoothing and computational complexity. In recent years, the fusion of multi-scale and residual structures has become a research hotspot. The dehazing network proposed by Yi et al. [39] enhanced the perception of haze distribution through adaptive feature selection. These advancements provide an innovative space for the proposal of dynamic multi-scale feature fusion residual modules [40–42]. Related studies have also extended dehazing and adverse-weather restoration to more complex scenarios, including nighttime haze removal [43–46] and general weather-degraded image restoration [47–51]. These works provide useful references for degradation-aware and multi-scale restoration.

### Models

We design a U-Net-based encoder-decoder architecture for the image defogging task, in which the main innovations are embodied in three custom modules: *Convolutional Squeeze and Excitation Attention (CSEA)*, *Nonlinear CSEA-Coupled Residual Block (NCCRB)*, and *Multi-scale Parallel Dilated Convolution Kernel NCCRB Block (MSPLCK-NCCRB)*.

**Fig 1:**
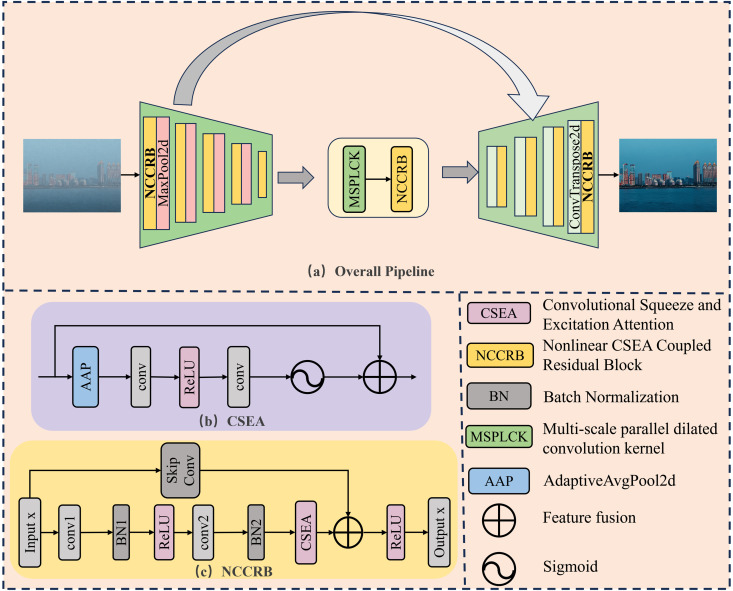
Overall architecture of the proposed DMSH-Net. The network adopts an encoder-bottleneck-decoder structure for depth-aware multi-scale dehazing. In the encoder and decoder, NCCRB is used for local residual feature refinement and detail preservation. At the bottleneck stage, MSPLCK-NCCRB aggregates multi-scale contextual information through parallel dilated convolutions to enhance the perception of depth-related haze variations. CSEA is embedded into NCCRB to recalibrate haze-relevant channel responses, forming a hierarchical restoration process from local feature enhancement to multi-scale context modeling.

Fig 1 depicts the overall framework, which exhibits a typical U-shaped topology with an encoding stage, a bottleneck module, and a decoding stage. In the encoder part, the network employs multiple NCCRBs to extract image features, which are downsampled following the max pooling layer. Each residual block can optionally incorporate CSEA. The features obtained from the encoder layer are sent to the bottleneck layer, where they are refined through the MSPLCK module. The use of dilated convolution increases the receptive field, thereby capturing multi-scale information of the image and further enhancing the dehazing effect. The decoder gradually restores image details through upsampling and NCCRB operations and fuses high-level and low-level features through residual connections to ensure the global consistency of the image. Finally, a convolutional layer outputs the dehazed image, which is normalized using the Sigmoid function. The entire network demonstrates excellent performance in the dehazing task, effectively restoring clear images.

### Convolutional squeeze and excitation attention

In the image dehazing task, the traditional SE attention mechanism faces two crucial challenges: feature compression distortion and static weight limitations. When the SE module compresses the spatial dimension through global average pooling, it results in the loss of high-frequency details. It fails to effectively handle the local feature differences of non-uniform fog distribution. Moreover, its channel re-scaling weights rely solely on global statistical information, lacking adaptability to dynamic fog concentration changes. This leads to insufficient suppression ability for areas with remaining haze. Therefore, we designed the CSEA attention module to retain spatial context information under different receptive fields, introduce non-linear activation to enhance feature interaction capabilities, retain the original features through residual connections, and make the design more lightweight.

As shown in Fig 1(b), we first perform global average pooling on each channel using adaptive average pooling, compressing the spatial dimension of the image to 1x1 to obtain the global average value of each channel. It can be expressed as


$$\begin{array}{c} A=\text{AdaptiveAvgPool2d}(X), \end{array}$$ (2)

where A represents the pooled image feature map, with dimensions (B, C, 1, 1), where each channel corresponds to the global average feature. Subsequently, the channel-wise attention map is computed using two successive 1×1 convolutional layers. Initially, a convolution operation reduces the channel dimension from *C* to *C*/16, followed by the ReLU activation to introduce non-linearity. The channel size is then restored to *C* using another 1×1 convolution. The formulation is given by


$$\begin{array}{c} Z=\sigma (W_{2}\cdot \text{ReLU}(W_{1}\cdot A)), \end{array}$$ (3)

where *W*_1_ and *W*_2_ denote the weight parameters of the two convolutional layers responsible for producing the attention response. Here, ReLU(·) applies the rectified linear unit activation, while σ(·) represents the Sigmoid function, which normalizes the output to the range [0, 1]. The resulting vector *Z* encodes the attention weights across all channels. Finally, the computed attention weights are applied to the input feature map through element-wise multiplication along the channel dimension. This operation produces a refined output feature map and can be formulated as


$$\begin{array}{c} Y=X⊙Z, \end{array}$$ (4)

where ⊙ denotes element-wise multiplication, and *Y* is the resulting feature map after weighting.

Through above adaptive mechanism, the output *Y* emphasizes more informative channels by enhancing their responses, while suppressing less relevant ones. Consequently, the CSEA module enables dynamic feature modulation according to the relative significance of each channel, thereby reinforcing critical information within the image.

### Nonlinear CSEA coupled residual block

As shown in Fig 1(c), it consists of two convolutional kernels, each followed by a batch normalization and an activation function. After the second batch normalization, the CSEA is introduced, followed by a skip connection and another activation.

First, the input image *X* undergoes a series of convolution operations, starting with two convolutional layers, followed by batch normalization and ReLU activation


$$\begin{array}{c} \left\{ \begin{array}{cc} {\hat{X}}_{1} & =\text{ReLU}(BN_{1}({\text{Conv}}_{1}(X))),\\ {\hat{X}}_{2} & =BN_{2}({\text{Conv}}_{2}({\hat{X}}_{1})), \end{array}\right. \end{array}$$ (5)

among them, *Conv*_1_ and *Conv*_2_ are convolution operations, using 3×3 kernels with padding 1. *BN*_1_ and *BN*_2_ are batch normalization layers. *X*_1_ and *X*_2_ represent the feature maps after convolution operations, processed through activation and normalization. When the CSEA attention mechanism is enabled during module initialization, the input image will pass through the CSEA module, which adaptively adjusts the channel features. The CSEA module works by applying weights to the input features to enhance important features and suppress unimportant ones. The formula is as


$$\begin{array}{c} Y={\hat{X}}_{2}⊙Z, \end{array}$$ (6)

Similar to traditional residual networks, the module contains a skip connection, where the input *X* is passed through a 1×1 convolution and added to the processed features. It can be expressed as


$$\begin{array}{c} \left\{ \begin{array}{c} I=\text{SkipConv}(X),\\ Y_{\text{final}}=Y+I, \end{array}\right. \end{array}$$ (7)

where *SkipConv*(*X*) is the operation where the input *X* is passed through a 1×1 convolution for dimension matching, and *Y*_final_ is the final output feature map. Finally, the output feature map *Y*_final_ is passed through a ReLU activation function to introduce non-linearity, producing the final result


$$\begin{array}{c} X_{\text{out}}=\text{ReLU}(Y_{\text{final}}), \end{array}$$ (8)

where *X*_out_ is the final output of the residual convolution block. This design combines residual learning with an adaptive attention mechanism, enabling the model to enhance the representation of important features in the image, while preserving fine details and avoiding the loss of information during the recovery process.

### Multi-scale parallel dilated convolution Kernel – NCCRB Block

**Fig 2:**
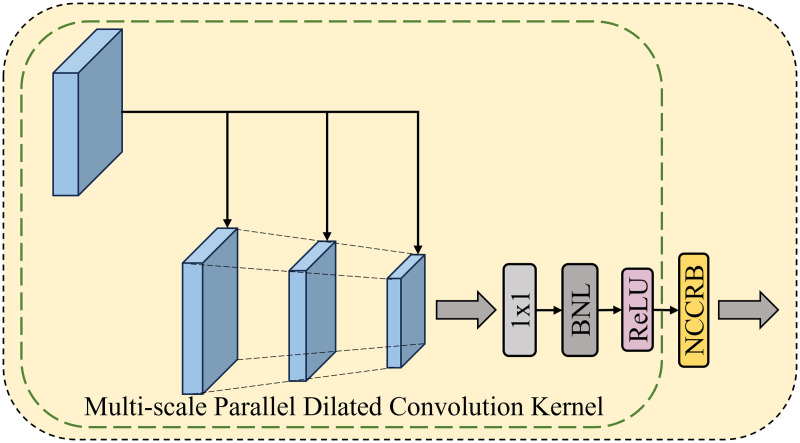
The Multi-scale Parallel Dilated Convolution Kernel NCCRB (MSPLCK-NCCRB) bottleneck module. MSPLCK-NCCRB performs parallel multi-scale processing to expand the receptive field, thereby adapting to the spatial disparities of haze between near and distant scenes. It enhances structural information in blurred regions and preserves texture details in clearer areas. The resulting features, enriched with multi-scale contextual information, are then forwarded to the decoder, improving the overall consistency and detail reconstruction of the dehazing results.

In this paper, we propose a new bottleneck module, Multi-scale Parallel Dilated Convolution Kernel NCCRB (MSPLCK-NCCRB), designed to address the spatially non-uniform haze distribution in real-world images-where distant regions typically exhibit low transmission and blurred structures, while near-field areas contain clearer textures yet are susceptible to over-enhancement artifacts. As shown in Fig 2, we adopt a parallel multi-scale receptive-field design that concurrently perceives structural features and haze density across varying depth regions. Specifically, dilated convolutions with different receptive fields are used to aggregate haze-related contextual cues at multiple spatial ranges, where larger receptive fields help capture long-range haze degradation in distant regions and smaller receptive fields help preserve local structures in near-field regions. By enabling unified modeling and enhancement of complex haze distributions, the module improves the robustness of dehazing networks across diverse scenes and enhances the accuracy of detail restoration.

The input image *X* passes through a multi-scale parallel dilated convolution kernel module to extract and fuse multi-scale features


$$\begin{array}{c} X_{\text{fuse}}=\text{MSPLCK}(X), \end{array}$$ (9)

where the *X*_fuse_ is the multi-scale features extracted and fused by the MSPLCK module.

The residual convolution block further processes *X*_fuse_ as


$$\begin{array}{c} Y=\text{NCCRB}(X_{\text{fuse}}), \end{array}$$ (10)

where the *Y* is the feature map processed by the residual convolution block, which includes convolution, batch normalization, ReLU activation, and optionally the CSEA attention.

Then, the output of MSPLCK, *X*_fuse_, and the feature map *Y* processed by NCCRB are fused as


$$\begin{array}{c} X_{\text{out}}=Y+X_{\text{fuse}}, \end{array}$$ (11)

following the processing, the output is obtained by residual connection and results in the final output *X*_out_. The result preserves the fine details of the image, while avoiding the nonlinear transformations that occur during early processing.

### Loss function

Mean Squared Error (MSE), or commonly referred to as the *L*_2_ loss, is frequently adopted as a loss function for single-image dehazing tasks. However, according to Lim et al. [52], training with the *L*_1_ loss typically leads to superior performance in terms of PSNR and SSIM compared to using the *L*_2_ loss in various image restoration scenarios. Motivated by this finding, the *L*_1_ loss is selected as the primary optimization criterion in our study. Although several existing dehazing approaches integrate additional loss terms, such as perceptual or generative adversarial (GAN) losses, our proposed method exclusively employs the *L*_1_ loss to maintain simplicity and effectiveness.

Let $\hat{I}$ represent the predicted image and *I* denote the corresponding ground truth. The *L*_1_ loss function can be mathematically expressed as


$$\begin{array}{c} {ℒ}_{L_{1}}=\frac{1}{N}\sum_{i=1}^{N}\left|{\hat{I}}_{i}-I_{i}\right|, \end{array}$$ (12)

where *N* indicates the total number of pixels in the image. This formulation penalizes the absolute difference between the predicted and ground truth pixel values, thereby promoting more stable and structurally consistent reconstructions.

In summary, we propose a U-shaped encoder-decoder network for image dehazing, centered on three innovative modules: CSEA, NCCRB, and MSPLCK-NCCRB. The encoder uses NCCRBs for feature extraction and CSEA for channel attention, while the bottleneck employs MSPLCK to expand the receptive field and integrate multi-scale context. We use Mean Absolute Error (MAE) loss for supervision, ensuring stable convergence and better edge preservation. This architecture integrates attention, residual learning, and multi-scale context modeling, achieving robust and accurate dehazing.

### Experiments

All experiments were conducted using Python 3.9 and the PyTorch framework on an NVIDIA Tesla V100S GPU with 32 GB memory. During training, all input images were resized to 256×256, and the network was optimized using Adam with an initial learning rate of $1\times 10^{-5}$ and the *L*_1_ loss.

### Experimental setting

The proposed method was trained and evaluated on both synthetic and real-haze datasets. Synthetic data were drawn from RESIDE, using ITS and OTS as training subsets and SOTS-Indoor and SOTS-Outdoor as test subsets. We also used RESIDE-6K, a paired synthetic dataset containing indoor and outdoor hazy clean image pairs. Real-world data included I-HAZE (35 indoor pairs), O-HAZE (45 outdoor pairs), and the HazyDet UAV-view benchmark with both natural and physically modeled haze. To further evaluate the real-world generalization performance of the proposed method under reference-free conditions, we additionally conducted experiments on the RTTS dataset, which contains real hazy images without paired haze-free reference images. For this evaluation, the model trained on RESIDE-6K was directly tested on RTTS without additional fine-tuning. Training and evaluation followed the official splits or a fixed random split.

We compared DMSH-Net with representative single-image dehazing methods from different categories, including prior-based, CNN-based, attention-based, multi-scale, transformer-style, and physics-guided approaches. Full-reference comparisons on RESIDE, RESIDE-6K, HazyDet, and I/O-HAZE were conducted against classical and recent deep models, such as DCP [10], DehazeNet [24], AOD-Net [25], GridDehazeNet [26], MSBDN [53], FFA-Net [28], DehazeFormer [54], C2PNet [55], RIDCP [56], and other representative methods listed in Tables 1–4. For the reference-free RTTS evaluation, we further included representative real-world dehazing methods, including PDN [57], MBDN [53], DH (DeHamer) [58], DAD [19], PSD [59], D4 [60], RIDCP [56], and DGUN [61]. To ensure clarity and reproducibility, the results of DMSH-Net were obtained using our implementation under the training and testing settings described above. For reproduced baselines, official implementations or released pretrained models were used whenever available, and the same evaluation scripts were adopted. For baselines that were not reproduced, the reported values were taken from the corresponding original papers or published benchmark protocols. In particular, the RTTS baseline results were collected from a published real-world dehazing evaluation protocol, while DMSH-Net was directly tested on RTTS using the RESIDE-6K-trained model under the same no-reference metrics.

To quantitatively evaluate the methods, we used Peak Signal-to-Noise Ratio (PSNR) and Structural Similarity Index (SSIM) as image quality metrics. For datasets with paired haze-free reference images, PSNR and SSIM were adopted as full-reference metrics and computed between the restored images and the corresponding ground-truth images. For the reference-free RTTS dataset, where ground-truth clear images are unavailable, we used three no-reference metrics, including FADE, BRISQUE, and NIMA. FADE and BRISQUE are lower-is-better metrics, while NIMA is a higher-is-better perceptual quality metric.

## Experimental results and discussion

We evaluate the proposed method through full-reference quantitative metrics, no-reference real-world assessments, ablation studies, and complexity analysis to comprehensively analyze its dehazing performance.

### Quantitative comparison

In terms of quantitative evaluation, Table 1 reports results on RESIDE, a synthetic hazy dataset covering indoor and outdoor scenes, where the proposed method attains higher average PSNR and SSIM. Table 2 presents results on RESIDE-6K, a larger-scale synthetic dataset, indicating good adaptability to large-scale synthetic data. Table 3 summarizes results on HazyDet, a detection-oriented dataset with real hazy images, where the method maintains strong PSNR and SSIM. Table 4 shows results on I/O-HAZE, a real indoor-outdoor dataset with spatially non-uniform haze, on which the method yields consistent improvements. To further evaluate the practical performance of the proposed method in real-world reference-free scenarios, Table 5 reports no-reference quantitative results on the RTTS dataset. Since RTTS does not provide paired haze-free ground-truth images, full-reference metrics such as PSNR and SSIM are not applicable. Therefore, FADE, BRISQUE, and NIMA are adopted for evaluation, where lower FADE and BRISQUE values indicate better haze removal and perceptual quality, while a higher NIMA value indicates better aesthetic quality. The proposed method achieves the best FADE score, suggesting effective haze-density reduction on real-world hazy images. Although RIDCP obtains better BRISQUE and NIMA values, DMSH-Net still shows competitive perceptual quality while achieving stronger haze suppression.

**Table 1:** The performance results of various methods on the RESIDE-IN and RESIDE-OUT datasets.

Method	RESIDE-IN		RESIDE-OUT	
	PSNR	SSIM	PSNR	SSIM
GCANet [27]	30.23	0.980	–	–
GridDehazeNet [26]	32.16	0.984	30.86	0.982
DCP [10]	16.62	0.818	19.13	0.815
DehazeNet [24]	19.82	0.821	24.75	0.927
UHD [62]	21.75	0.878	26.48	0.942
AOD-Net [25]	20.51	0.816	24.14	0.920
MSBDN [53]	33.67	0.985	33.48	0.982
PFDN [63]	32.68	0.976	–	–
FFA-Net [28]	36.39	0.989	33.57	0.984
AECR-Net [64]	37.17	0.990	–	–
PMNet [65]	38.41	0.990	34.74	0.985
MAXIM [66]	38.11	0.991	34.19	0.985
DeHamer [58]	36.36	0.988	35.18	0.986
UDN [67]	38.62	0.991	34.93	0.987
MixDehazeNet [68]	42.62	**0.997**	36.50	0.986
Ours	**42.96**	0.995	**43.98**	**0.994**

**Table 2:** The performance results of various methods on the RESIDE-6K dataset.

Method	RESIDE-6K	
	PSNR	SSIM
GCANet [27]	25.09	0.944
GridDehazeNet [26]	25.86	0.944
DCP [10]	17.88	0.816
DehazeNet [24]	21.02	0.870
AOD-Net [25]	20.27	0.855
MSBDN [53]	28.56	0.966
PFDN [63]	28.15	0.962
FFA-Net [28]	29.96	0.973
AECR-Net [64]	28.52	0.964
MixDehazeNet [68]	30.18	0.973
Ours	**41.94**	**0.988**

**Table 3:** The performance results of various methods on the HazyDet dataset.

Method	HazyDet	
	PSNR	SSIM
GridDehaze [26]	15.66	0.813
MixDehazeNet [68]	17.52	0.843
DSANet [69]	21.01	0.851
FFA-Net [28]	21.25	0.898
DehazeFormer [54]	19.53	0.832
gUNet [70]	21.59	0.842
C2PNet [55]	21.31	0.832
DCP [10]	19.48	0.844
RIDCP [56]	19.03	0.808
Ours	**27.10**	**0.966**

**Table 4:** The performance results of various methods on the I/O-HAZE dataset.

Method	I/O-Haze	
	PSNR	SSIM
DCP [10]	15.90	0.713
ROP+ [71]	13.17	0.723
SLP [72]	15.56	0.736
USID-Net [73]	16.40	0.665
RIDCP [56]	17.95	0.754
ODCR [74]	16.93	0.734
UCL [75]	16.97	0.777
YOLY [76]	16.51	0.739
Ours	**23.21**	**0.801**

**Table 5:** The no-reference performance results of various methods on the RTTS dataset.

Method	RTTS		
	FADE↓	BRISQUE↓	NIMA↑
Hazy	2.484	36.642	4.483
PDN [57]	0.876	30.811	4.464
MBDN [53]	1.363	27.672	4.529
DH [58]	1.895	33.862	4.522
DAD [19]	1.130	32.241	4.312
PSD [59]	0.920	27.713	4.598
D4 [60]	1.358	33.210	4.484
RIDCP [56]	0.944	**17.293**	**4.965**
DGUN [61]	1.111	27.968	4.653
Ours	**0.698**	29.438	4.435

Overall, these quantitative results suggest that the proposed method achieves consistent restoration performance across both full-reference paired benchmarks and reference-free real-world hazy scenes.

### Ablation study

Ablation studies are conducted to analyze the contribution of different components to the overall dehazing performance. The proposed DMSH-Net mainly contains three key designs: the CSEA attention module, the NCCRB nonlinear residual block, and the MSPLCK-NCCRB multi-scale bottleneck module. To verify the effectiveness of these components, we analyze the model from five aspects: overall component combination, attention mechanism replacement, residual block replacement, bottleneck structure replacement, and model complexity.

It should be noted that the overall component-wise ablation study follows the same training configuration as the final model and is trained for 2000 epochs to sufficiently evaluate the performance of different module combinations. The subsequent fine-grained structure replacement experiments are conducted under a unified controlled setting, where all variants use the same data split, input size, optimizer, learning rate, and loss function. Specifically, the input images are resized to 256×256, Adam is adopted as the optimizer, the initial learning rate is set to $1\times 10^{-5}$, and the *L*_1_ loss is used as the optimization objective. Each model in the fine-grained ablation experiments is trained for 300 epochs. Therefore, the absolute PSNR values across different tables are not directly compared; instead, we mainly focus on the performance differences among variants under the same experimental setting.

First, we conduct an overall component-wise ablation study to analyze the contribution of CSEA, NCCRB, and MSPLCK-NCCRB. The results are reported in Table 6. Compared with the basic U-shaped encoder–decoder structure, introducing MSPLCK improves the PSNR from 35.51 dB to 36.89 dB, indicating that multi-scale feature modeling is beneficial for image dehazing. When NCCRB is further incorporated, the PSNR and SSIM increase to 38.30 dB and 0.98, respectively, showing that this structure further enhances feature representation. When all three modules are used together, the model achieves the best performance, with 41.94 dB PSNR and 0.99 SSIM, which verifies the complementary effect among these components.

**Table 6:** Component-wise ablation study on the RESIDE-6K dataset.

Method	MSPLCK	NCCRB	CSEA	PSNR↑	SSIM↑
Base	×	×	×	35.51	0.97
Base + MSPLCK	√	×	×	36.89	0.97
Base + MSPLCK-NCCRB	√	√	×	38.30	0.98
**Base + MSPLCK-NCCRB + CSEA**	√	√	√	**41.94**	**0.99**

To further analyze the role of CSEA, we compare it with several commonly used attention mechanisms, including SE, ECA, and CBAM. The results are shown in Table 7. Compared with the variant without attention, SE, ECA, and CSEA all bring noticeable performance improvements, indicating that channel feature recalibration is beneficial for the dehazing task. Among them, CSEA achieves the highest PSNR of 28.39 dB and maintains a competitive SSIM of 0.95, outperforming the other attention mechanisms in terms of restoration accuracy.

**Table 7:** Ablation comparison of different attention mechanisms on the RESIDE-6K dataset.

Attention module	PSNR↑	SSIM↑
Without attention	27.39	0.94
SE	28.32	0.95
ECA	28.31	0.95
CBAM	27.56	0.94
**CSEA**	**28.39**	**0.95**

Next, we analyze the effectiveness of NCCRB. For this purpose, we compare four structures: Plain Conv Block, Basic Residual Block, Residual Block + CSEA, and NCCRB. The results are reported in Table 8. Compared with the plain convolutional block, introducing residual connections improves the PSNR from 27.29 dB to 27.37 dB, indicating that residual learning helps preserve structural information. When CSEA is further added, the model performance is further improved. The complete NCCRB achieves the highest PSNR of 28.39 dB, while obtaining a comparable SSIM of 0.95. This demonstrates that NCCRB provides a favorable overall performance.

**Table 8:** Ablation comparison of different residual block designs on the RESIDE-6K dataset.

Block design	Residual connection	CSEA	PSNR↑	SSIM↑
Plain Conv Block	×	×	27.29	0.94
Basic Residual Block	√	×	27.37	0.92
Residual Block + CSEA	√	√	28.28	0.94
**NCCRB**	√	√	**28.39**	**0.95**

We then analyze the role of the MSPLCK-NCCRB bottleneck module. Since haze distribution usually exhibits obvious spatial scale variations, the receptive-field design in the bottleneck layer is important for dehazing performance. Table 9 compares the standard convolutional bottleneck, single dilated convolutional bottleneck, multi-scale dilated bottleneck, and MSPLCK-NCCRB bottleneck. The results show that the standard convolutional bottleneck obtains relatively lower performance, while introducing dilated convolution improves the results, indicating that enlarging the receptive field is helpful for modeling haze-related contextual information. MSPLCK-NCCRB achieves a PSNR of 28.38 dB and maintains a competitive SSIM of 0.95, demonstrating its good overall performance.

**Table 9:** Ablation comparison of different bottleneck structures on the RESIDE-6K dataset.

Bottleneck design	Dilation setting	PSNR↑	SSIM↑
Standard Conv Bottleneck	1	28.30	0.95
Single Dilated Conv Bottleneck	2	**28.40**	0.95
Multi-scale Dilated Bottleneck	1, 2, 3	28.33	0.94
**MSPLCK-NCCRB Bottleneck**	1, 2, 3 + NCCRB	28.38	**0.95**

Finally, we report the complexity of different model variants to analyze the relationship between performance improvement and computational cost. Table 10 presents the number of parameters, computational cost measured by GMACs, floating-point operations, average inference time, and FPS. Here, GMACs denote the multiply-accumulate operations for a single 256×256 input image, and FLOPs are approximated as FLOPs≈2×MACs.

**Table 10:** Complexity comparison of different DMSH-Net variants.

Method	Params (M)↓	GMACs↓	FLOPs (GFLOPs)↓	Runtime (ms)↓	FPS↑
Baseline U-Net	10.40	41.27	82.54	7.86	127.16
Base + MSPLCK	15.91	46.91	93.82	10.15	98.48
Base + MSPLCK-NCCRB	32.95	64.36	128.72	15.05	66.45
Full DMSH-Net	33.04	64.38	128.75	16.25	61.55

As MSPLCK, NCCRB, and CSEA are introduced, the model complexity gradually increases. The complete DMSH-Net contains 33.04 M parameters and requires 64.38 GMACs, corresponding to 128.75 GFLOPs. Its average inference time is 16.25 ms, with an FPS of 61.55. Notably, compared with Base + MSPLCK-NCCRB, introducing CSEA only increases the number of parameters by 0.09 M and the computational cost by 0.02 GMACs, while bringing stable performance improvements. Combining the results from Table 6 to Table 9, the additional computational cost can be effectively translated into performance gains, indicating that DMSH-Net achieves a reasonable balance between restoration quality and model complexity.

Overall, the ablation experiments verify the effectiveness of each proposed component. CSEA enhances channel feature selection, NCCRB improves feature representation and structural recovery, and MSPLCK-NCCRB strengthens contextual modeling through multi-scale receptive fields. These components work together to enable DMSH-Net to achieve better performance in detail restoration, structure preservation, and complex haze removal.

## Conclusion

In this paper, we proposed a new dehazing method that incorporates key modules such as CSEA, NCCRB, and MSPLCK-NCCRB Bottleneck to improve image quality in complex hazy conditions. First, a convolutional squeeze-and-excitation attention (CSEA) module is designed to replace fully connected layers with convolutional operations and global average pooling, jointly capturing channel dependencies and spatial context to enhance fine detail preservation. Second, a nonlinear CSEA-coupled residual block (NCCRB) integrates nonlinear activations with residual learning to strengthen local feature representation and adapt to varying haze densities. In addition, a multi-scale dilated convolution bottleneck is employed to model depth-related spatial haze variations across multiple receptive fields, effectively addressing spatially non-uniform haze. Collectively, these components endow DMSH-Net with robust performance, improved restoration accuracy, structural similarity, and robustness across synthetic and real-world dehazing benchmarks.

Despite the method’s overall stable performance, there remains aspects to improve efficiency and cross-domain robustness, and future work will proceed along the following directions:

- Because the network adopts a fusion design with multiple submodules, inference time is relatively long. Future work will optimize operators and computation paths to pursue higher efficiency without sacrificing restoration quality.
- The future work will explore self-supervised and unpaired learning to reduce reliance on synthetic data, and strengthen the coupling between physical priors and data priors to enhance interpretability, cross-domain generalization, and deployment readiness.
